# Bilingualism and creativity: Benefits from cognitive inhibition and cognitive flexibility

**DOI:** 10.3389/fpsyg.2022.1016777

**Published:** 2022-11-03

**Authors:** Tiansheng Xia, Yi An, Jiayue Guo

**Affiliations:** School of Art and Design, Guangdong University of Technology, Guangzhou, China

**Keywords:** bilingualism, cognitive inhibition, cognitive flexibility, convergent thinking, divergent thinking, creativity

## Abstract

Bilingualism has been shown to be associated with creativity, but the mechanisms of this association are not very well understood. One possibility is that the skills that bilinguals use in switching back and forth between languages also promote the cognitive processes associated with creativity. We hypothesized that high-proficient Chinese-English bilinguals would show higher convergent and divergent thinking than low-proficient bilinguals, with the differences being mediated by cognitive inhibition and cognitive flexibility, respectively. Chinese university students (*N* = 54) were classified as high-proficient (*n* = 27) and low-proficient (*n* = 27) bilinguals based on their performance on the National English Test for College Students. As expected, group comparisons showed that the high-proficient group had higher scores on the Remote Associates Test (RAT, convergent thinking) and the Torrance Test of Creative Thinking (TTCT, divergent thinking). Also as expected, the association between bilingualism and convergent thinking was mediated by scores on a Stroop task (cognitive inhibition), and the association between bilingualism and divergent thinking was mediated by scores on a More-odd shifting task (cognitive flexibility). These findings suggest that bilingual learning can promote the development of different components of creativity through stronger cognitive inhibition and cognitive flexibility. The results provide empirical evidence for the relationship and mechanism between bilingual learning and creativity.

## Introduction

Many studies have found that bilingualism is positively correlated with creativity (Kharkhurin, 2010a,b Hommel et al., 2011; Lee and Kim, 2011; Leikin, 2013; Leikin and Tovli, 2014). However, there is no agreement on which aspects of creativity are predicted by bilingualism, or the reason for these associations. One possibility is that the skills that bilinguals use in switching back and forth between languages also promote the cognitive processes associated with creativity.

Creativity refers to the ability to produce novel, unique and valuable products or ideas (Sternberg and Lubart, 1996), mainly including divergent thinking and convergent thinking (Guilford et al., 1967). Divergent thinking is the ability to produce a variety of possible answers or different solutions to a problem and is measured by fluency, flexibility and originality (Guilford, 1968; Kim, 2006; Runco, 2008; Kharkhurin, 2017). Convergent thinking is the ability to use existing knowledge or traditional methods to analyze given information and obtain the best answer (Runco, 2004; Cropley, 2006; Gabora, 2010).

Many studies have shown that bilinguals show greater creativity than monolinguals (Leikin et al., 2020). Compared to monolingual groups, Korean-American college students showed higher general creativity (Lee and Kim, 2011); Hebrew-Russian children age 4–6 showed higher mathematical creativity (Leikin, 2013) and higher originality and nonverbal creativity (Leikin and Tovli, 2014). However, there have also been different results. In one study, Russian-English bilinguals showed higher nonverbal creativity than monolinguals, but they showed weaker verbal creativity (Kharkhurin, 2010a). Bilingualism was also found to promote higher convergent but not divergent thinking, in a study of Dutch–German bilinguals (Hommel et al., 2011). Another consideration is that the association between bilingualism and creativity may vary based on the cultural context. Kharkhurin (2010b) found that American Russian-English bilinguals showed significantly higher generative capacity than English monolinguals, but Iranian Farsi-English bilinguals did not. Iranian Farsi-English bilinguals had significantly higher innovative capacity than Farsi monolinguals, whereas American bilinguals and monolinguals did not differ on this measure of creativity.

The link between bilingualism and creativity might be explained in part by cognitive control (Bialystok et al., 2005; Bialystok, 2011; Sampedro and Peña, 2019). Cognitive control refers to the ability to regulate and monitor ongoing behaviors to achieve goals in novel ways in changing and emergent situations (Ridderinkhof et al., 2004). It involves being able to flexibly change plans and behaviors, suppress inappropriate behaviors, monitor and resolve conflicts, and detect and learn from mistakes (Mackay et al., 2004; van Gaal et al., 2011). Cognitive control consist of three parts (Diamond, 2013), namely cognitive inhibitory control, cognitive flexibility, and working memory (Miyake et al., 2000; Lehto et al., 2003). In the current study, we focus on cognitive inhibitory control and cognitive flexibility as mediators of the association between bilingualism and creativity. Bilingual learning has been shown to be associated with the development of cognitive inhibition and cognitive flexibility (Bialystok, 2011). In addition, cognitive inhibition and cognitive flexibility have been shown to be closely related to convergent and divergent thinking, respectively (Baas et al., 2008; Chermahini and Hommel, 2012).

As in the present study, Hommel et al. (2011) explored the relationship between bilingualism and creativity using measures of divergent thinking and convergent thinking rather than creativity as a whole. The researchers suggested that bilingual learning biased competition among cognitive representations. Top-down processes then create a control state that is conducive to convergent thinking. By contrast, divergent thinking benefits from a cognitive control state that involves a minimum of top-down bias. Hommel et al. (2011) research is highly relevant to the current study. However, unlike their view of cognitive control as a state, we studied two components of cognitive control: cognitive inhibition and cognitive flexibility.

The first mechanism we test in the current study is cognitive inhibitory control as a mediator of the association between bilingualism and convergent thinking. Processing a new language requires the inhibition of the main language, which may improve cognitive inhibition over time (Green, 1998; Bialystok, 2015). Bilingual processing is also thought to require selective attention to the relevant language and suppression of the irrelevant one (Cortes et al., 2019). Bialystok et al. (2008) used a spatial Stroop task to compare the performance of bilinguals and monolinguals, and found that bilinguals had significantly better performance in the interference effect of inhibition. Recent studies also found that high-proficient bilinguals tend to have higher cognitive inhibition than low-proficient bilinguals (Tran et al., 2019; Xie and Zhou, 2020).

Cognitive inhibition is closely related to convergent thinking (Baron-Cohen et al., 2003; Ridderinkhof et al., 2004). Bristol and Viskontas (2006) asserted that cognitive inhibition prevents the creation of useless connections, so that individuals can more effectively apply cognitive resources to creative problem solving. For example, White and Shah (2006) used distance association tasks and found that participants who showed greater inhibition of semantically interfering items performed better on convergent thinking tasks. Similarly, Koppel and Storm (2014) demonstrated that cognitive inhibition benefits creative problem solving on remote associative tasks. Therefore, we hypothesized that bilingual learning can promote the development of cognitive inhibition an in turn, greater convergent thinking.

The second mechanism we test in the current study is cognitive flexibility as a mediator of the association between bilingualism and divergent thinking. Bilingual learning has been shown to be associated with higher cognitive flexibility (Bialystok and Senman, 2004). Cognitive flexibility refers to an individual’s ability to switch between different task states and mental stereotypes (Miyake et al., 2000). Early bilingualism is associated with developmental advantages in non-verbal tasks requiring cognitive flexibility (Bialystok and Senman, 2004; Carlson and Meltzoff, 2008). Prior and Macwhinney (2010) found that compared to monolingual college students, bilingual students activated a switch task more quickly in response to the prompt. Bilingual students were also more able to overcome any interference caused by the performance of the previous task. That is, the switching cost of bilingual students was less than that of monolingual students. Recent studies have also found that individuals increase cognitive flexibility in learning and mastering a foreign language (Javan and Ghonsooly, 2018).

There are conceptual and empirical reasons to conclude that cognitive flexibility in turn is associated with divergent thinking (Kharkhurin, 2017). Baas et al. (2008) asserted that creativity is the product of innovative processing, which includes flexibility in information processing and divergent cognitive thinking. That is, when individuals are more flexible and divergent in their thinking, they are more creative (Nijstad et al., 2010; Chen et al., 2019). Cognitive flexibility may moderate to strong positive correlations with creativity (Chen et al., 2022). Therefore, we hypothesize that bilingual learning can promote the development of cognitive flexibility and thus promote the improvement of divergent thinking.

Though several studies have shown that bilingualism has a specific, positive effect on creativity and cognitive control, other studies have shown opposite results (Lehtonen et al., 2018; Lange et al., 2020; Booton et al., 2021). Booton et al. (2021) used a linguistic task, figural task, and ideational task to measure divergent thinking in a group of 111 bilingual and monolingual children. The results showed that there were no differences between the bilingual and monolingual children across any of the divergent thinking tasks. Lange et al. (2020) used Bayesian statistics to analyze performance on the alternative uses task (AUT). They concluded that bilingualism offered no advantage for creativity, and suggested that the mixed results in the literature were likely due to a high prevalence of false positives, or statistical noise.

However, some researchers have questioned whether there is a bilingual advantage for creativity and cognitive control because most of the evidence showing an advantage is based on the use of the AUT to measure creativity (Hommel et al., 2011; Lange et al., 2020). A recent study found that preadolescent children with a high level of bilingualism performed better on figural creativity tasks than those with a low level of bilingualism, but there was no group difference on verbal creativity tasks (Sampedro and Peña, 2019). In addition, a recent study found that balanced bilinguals performed better on the Torrance Tests of Creative Thinking (TTCT) and Remote Associates Test (RAT) than non-balanced bilinguals, but there was no significant group difference in executive functions (Leikin et al., 2020). Other research found that balanced bilinguals had a significant advantage over non-balanced bilinguals on most of the measures of metacognitive ability (e.g., planning, monitoring, and the use of metacognitive strategies) (Abu Rabia, 2019).

Leikin et al. (2020) asserted that one possible reason for the inconsistent results across studies has to do with measurement. Researchers conceptualize creativity differently and use different measures consistent with these conceptualizations (Booton et al., 2021). For example, creativity has been measured using tests of divergent thinking (Lange et al., 2020; Booton et al., 2021), verbal creativity tasks (Lange et al., 2020), and figural creativity tasks (Sampedro and Peña, 2019). In the current study we tested which of these various pieces of evidence can be explained by the mediating effect of cognitive control. Based on previous studies, we assume that bilingualism promotes cognitive control, and cognitive control in turn is associated with creativity. We pose two hypotheses. First, Chinese participants who are high-proficient in Chinese-English bilingualism will show higher convergent and divergent thinking than those who are low-proficient. Second, the association bilingualism and convergent thinking will be mediated by cognitive inhibition, and the association between bilingualism and divergent thinking will be mediated by cognitive flexibility.

## Materials and methods

### Participants

Fifty-four undergraduate and graduate students at Guangdong University of Technology participated in this experiment. The participants were divided into two groups according to whether they had passed the CET-4 and CET-6 tests (National English Test for College Students), which can test an individual’s general English proficiency through tasks including listening, reading comprehension, and writing (Fan et al., 2012). One group was labeled high-proficient English-Chinese bilinguals (*n* = 27); all of the students in this group had passed the CET-6. There were 10 men and 17 women in this group, with an average age of 21.9 ± 2.1 years. The other group was labeled low-proficient bilinguals (*n* = 27); all of the students had failed the CET-4. There were 13 men and 14 women in this group, with an average age of 22.1 ± 2.5 years. Self-assessment was used to estimate their English proficiency, and there was a significant difference in English proficiency between the two groups, *p* < 0.05. All participants reported that they had normal visual acuity or corrected visual acuity, and they were unfamiliar with the purpose of the study. All participants signed a written informed consent form prior to the formal study. At the end of the experiment, the participants received CNY 40 to thank them for their help.

### Procedure

The 60-min study was conducted in a laboratory setting. Participants were asked to conduct a self-assessment using the Language History Questionnaire, and then they completed the study tasks to assess their convergent thinking (RAT), divergent thinking (TTCT), cognitive inhibition (Stroop task) and cognitive flexibility (More-odd shifting task). The tasks were completed in random order. There was a 1–3 min break between each task. To ensure that all participants were able to follow the experimental procedures, all instructions were given in Chinese, the participants’ first language.

#### Language history questionnaire

The Language History Questionnaire has been shown to be an effective tool to assess the language ability of multilingual or second language learners (Li et al., 2020). The validity and reliability of the LHQ have been tested in a number of studies that showed correlations between LHQ results with other behavioral tests (Yang et al., 2015; Calvo et al., 2016). The proficiency module of the LHQ was used to measure participants’ bilingual ability, which provided one aggregated score based on participants’ self-rating of their proficiency in different components of a language (e.g., *Question: Rate your current ability in terms of listening, speaking, reading, and writing in each of the languages you have studied or learned*).

#### Measures RAT (convergent thinking)

The Remote Associates Test (Mednick, 1962) was used to test convergent thinking. An example in English would be the presentation of “gear,” “egg,” and “strong.” A correct answer would be “head,” as it can be combined with all of the three words to create new, reasonable words (“headgear,” “egghead,” “headstrong”). Given the familiarity of the language, we adopted a version of the RAT in Chinese (Xia et al., 2016). In Chinese, an example would be three original words of “巧,” “术,” and “科” to which “技” could be added either at the beginning or end to create three new reasonable words, “技巧,” “技术,” and “科技.” There were 58 trials. Participants were given 20 min to complete the task, and the number of correct answers within that time limit was the participant’s final score.

#### Torrance test of creative thinking (divergent thinking)

We used the Torrance Test of Creative Thinking (TTCT, Torrance, 1972) to assess divergent thinking. The task had three parts in which participants were asked to draw pictures based on known lines. Each part had 10 min to finish the questions. Judges rated the participants’ creativity on four indicators: fluency (produce the idea of the total); flexibility (number of categories or topics used by participants); elaboration (amount of detailed information provided); and originality (how unique a response is compared with other samples or populations). The participant received five ratings, including a rating for each indicator of creativity and a total TTCT rating. Each rating was made on a seven-point scale (1 = very dissatisfied; 7 = very satisfied). The judges were four students earning doctorates in design. The Kendall’s *W* coefficient for the TTCT was.031, *p* < 0.001, indicating that there was good inter-rater reliability.

#### Stroop task (cognitive inhibition)

The Stroop task is an effective method to measure cognitive inhibition (Groborz and Necka, 2003; Edl et al., 2014). It requires individuals to judge the meaning or color of words. In general, the reaction time is slower and the accuracy is lower when the color of words is incongruent with the meaning of words, which is referred to as a conflict effect (Stroop, 1935). The size of the Stroop effect is thought to reflect cognitive inhibition; individuals with high cognitive inhibition are more likely to resolve conflict and thus show a smaller Stroop effect (Edl et al., 2014).

The experiment was programmed using E-Prime 3.0, and the stimuli were presented on a LENOVO computer screen. The computer was equipped with a 24-inch LED display with a resolution of 1,024 × 768 and a refresh rate of 59 Hz. The participants were seated 45 cm from the screen. The stimulus materials were two Chinese one-character color words (“red” and “green”) in two different font colors (red and green) on a white background. When the color and the meaning of the word were congruent (e.g., “red” written in red), the trial was regarded as a congruent trial; when the color and the meaning of the word were incongruent (e.g., “red” written in green), the trial was regarded as an incongruent trial. Participants were asked to respond with the font color and to ignore the meaning of the word. They responded using a computer keyboard with their left and right index fingers. For example, they needed to respond to a red word by pressing the “J” key and respond to a green word by pressing “F.” The mapping between color and response key was balanced across participants. The task included 16 practice trials and 96 test trials that included 48 congruent trials and 48 incongruent trials. In each trial, there was a “+” fixation point presented for 500 ms, followed by a stimulus for 1,000 ms.

#### More-odd shifting task (cognitive flexibility)

Cognitive flexibility was measured using the more-odd shifting task (Salthouse et al., 1998; Chen et al., 2014). In this task, a numeric digit was presented on the screen and the participant was asked to make decisions about the digit based on combinations of the digit’s attributes in terms of color, magnitude, and whether the digit was odd or even. The digital stimulus font was 70-point Times New Roman with a white background. A random digit from 1 to 9 (excepting 5), randomly colored green or black, was presented on the screen. Participants were asked to respond to the stimulus by pressing the “J,” “K,” “D,” or “F” key on the keyboard.

There were two kinds of rules based on the color of the digit. According to rule A, if the digit was green, participants were asked to press “J” if the digit was odd, or press “K” if the digit was even. According to rule B, if the number was black, participants were asked to press “D” if the digit was larger than 5, or press “F” if the digit was smaller than 5. In each trial, the center of the screen first showed the “+” fixation point for 500 ms, followed by the digit for 1,200 ms. The participants’ response had to be given within 1,200 ms, after which the stimulus disappeared and there was a blank screen for 500 ms and the next trial began. To ensure participants understood and remembered the task rules, practice blocks included 16 trials before the formal experiment was conducted and the criterion was met. If the participants’ accuracy exceeded 80% in the practice block, they could continue with the formal experiment; otherwise they needed to continue to practice until they reached the criterion.

There were 64 trials in the formal experiment. We recorded the participants’ reaction times and accuracy during the more-odd shifting task. When the previous trial followed the same rule as the current trial (they were congruent), participants could respond without task shifting. When the previous trial followed a different rule from the current trial (they were incongruent), participants responded according to the shifting rule. Because the processing of task shifting requires a cognitive cost, the reaction time is usually slower and accuracy is lower in the incongruent condition compared to the congruent condition (Chen et al., 2014). The size of the difference between congruent and incongruent trials in the more-odd shifting task was considered to reflect cognitive flexibility. Previous research has shown that individuals with high cognitive flexibility experience less cost when switching tasks, and thus show less conflict effects in the more-odd shifting task (Cui et al., 2022).

## Results

The score of the National English Test for College Students was used to assign participants to the high-proficient and low-proficient groups. The self-assessment English score of the high-proficient group (*M* = 4.11, SD = 0.97) was significantly higher than that of the low-proficient group (*M* = 3.51, SD = 0.98), *F* (1, 52) = 4.99, *p* = 0.030, *ŋ_p2_* = 0.088.

Table 1 shows the average scores for the measures of creativity in the high-proficiency and low-proficiency groups. The high-proficient group scored higher than the low-proficient group on the RAT, *F*(1, 52) = 9.40, *p* = 0.003, *ŋ_p2_* = 0.153; the TTCT test overall score, *F*(1, 52) = 5.90, *p* = 0.019, *ŋ_p2_* = 0.102; and the TTCT subscale scores for flexibility, *F*(1, 52) = 6.10, *p* = 0.017, *ŋ_p2_* = 0.105, and elaborateness, *F*(1, 52) = 12.95, *p* < 0.001, *ŋ_p2_* = 0.199. However, there was no significant difference in TTCT fluency or originality between the two groups (*p_s_* > 0.05).

**Table 1:** Mean scores and standard deviation (in brackets) on the Remote Associates Test (RAT), Torrance Tests of Creative Thinking (TTCT), Stroop Task, and More-odd shifting Task by proficient groups.

Sample	High-proficient	Low-proficient
*N* (F:M)	27 (17:10)	27 (14:13)
Age	21.9 (2.1)	22.1 (2.5)
RAT^*^	27.38 (4.02)	23.80 (4.53)
TTCT Fluency	4.59 (0.73)	4.31 (1.03)
TTCT Flexibility^*^	4.39 (0.57)	3.90 (0.87)
TTCT Originality	4.23 (0.67)	3.86 (0.76)
TTCT Elaboration^**^	3.71 (0.69)	3.08 (0.71)
TTCT Grand Mean^*^	4.22 (0.52)	3.82 (0.71)
Cognitive Inhibition (ACC)	−0.01 (0.07)	0.01 (0.07)
Cognitive Inhibition (RT)^*^	19.27 (42.91)	42.09 (36.74)
Cognitive Flexibility (ACC)	−0.01 (0.20)	−0.04 (0.18)
Cognitive Flexibility (RT)^*^	53.67 (73.63)	98.74 (59.61)

* *p* < 0.05 and

** *p* < 0.01.

RAT: Remote Associates Task; TTCT: Torrance Test of Creative Thinking; ACC: accuracy; RT: reaction time.

For the Stroop task and the more-odd shifting task, correct response data outside the range of three standard deviations from the mean in each condition were considered outliers. In total, 0.8% of the trials in the Stroop task and 1.2% of the trials in the more-odd shifting task were excluded. The Stroop effect was calculated by comparing the reaction time (RT) and accuracy between the incongruent and congruent conditions, which was considered the index of cognitive inhibition. As shown in Table 1, for RT, we found that the Stroop effect was significantly smaller in the high-proficient group than in the low-proficient group, *F*(1, 52) = 4.41, *p* = 0.041, *ŋ_p2_* = 0.078. For accuracy, there was no significant difference between the two groups, *F*(1, 52) = 1.53, *p* = 0.221, *ŋ_p2_* = 0.029.

Similarly, the conflict effect in the more-odd shifting task was calculated by comparing RT and accuracy between the incongruent and congruent conditions, and the size of the conflict effect was considered the index of cognitive flexibility. The results showed that for RTs, the size of the conflict effect (53.67) of the high-proficient group was also significantly smaller than that of the low-proficient group (98.74), *F*(1, 52) = 6.11, *p* = 0.017, *ŋ_p2_* = 0.105. There was no significant difference in the accuracy between the two groups, *F*(1, 52) = 0.27, *p* = 0.609, *ŋ_p2_* = 0.005.

We calculated correlation coefficients among the study variables (see Table 2). Because for accuracy, the conflict effect was not significant, we used RT (the Stroop effect) as the index of cognitive inhibition. Similarly, We the RT (the conflict effect) in the more-odd shifting task as the index of cognitive flexibility. The grand mean in the TTCT test was used as the index of TTCT (divergent thinking). The results showed that there were no gender differences in any of the study variables, and age was not significantly correlated with any of the study variables (*p_s_* > 0.05). As expected, cognitive inhibition was significantly correlated with RAT (*r* = −0.332, *p* = 0.014), but not with TTCT (*r* = −0.258, *p* = 0.060). Cognitive flexibility was significantly correlated with TTCT (*r* = −0.289, *p* = 0.034), but not with RAT (*r* = 0.005, *p* = 0.969). The measures of RAT were not significantly correlated with TTCT (*r* = 0.215, *p* = 0.119). The results provide initial evidence of our hypotheses.

**Table 2:** Correlation matrix among variables.

	1	2	3	4	5	6
1. Gender	–					
2. Age	0.05	–				
3. RAT	0.11	0.14	–			
4. TTCT	0.22	−0.02	0.22	–		
5. Cognitive inhibition	0.10	−0.11	−0.33^*^	−0.26	–	
6. Cognitive flexibility	−0.18	−0.04	0.01	−0.29^*^	0.22	–

* *p* < 0.05.

RAT: Remote Associates Task; TTCT: Torrance Test of Creative Thinking.

To further explore the relationships among variables, participants’ proficiency score on the LHQ was regarded as the index of bilingual ability, and the size of the conflict effect on RT in the Stroop task and RT in the more-odd shifting task were regarded as the index of cognitive inhibition and cognitive flexibility, respectively. The SPSS PROCESS macro (Hayes, 2017) Model 4 was used to conduct two mediation analyses. The mediation effect was obtained by the corrected Bootstrap method with 5,000 samples. The mediation effect is considered significant at *p* < 0.05 if the 95% CI does not include 0.

First, cognitive inhibition was tested as a mediator in the relationship between bilingualism and convergent thinking. There was a significant direct effect of bilingualism on convergent thinking (*b* = 0.58, SE = 1.14, *p* = 0.023). In the mediated pathway, bilingual ability negatively predicted cognitive inhibition (*b* = −0.55, SE = 10.87, *p* = 0.041), and cognitive inhibition negatively predicted convergent thinking (*b* = −0.36, SE = 0.01, *p* = 0.006). The indirect effect of cognitive inhibition in the association between bilingualism and convergent thinking was 0.91, and its 95% confidence interval was [0.005, 0.524]. The indirect effect accounted for 25.5% of the total effect (*b* = 3.57, SE = 1.17, *p* = 0.003).

Second, cognitive flexibility was tested as a mediator in the relationship between bilingualism and divergent thinking. In the mediated pathway, bilingual ability negatively predicted cognitive flexibility (*b* = −0.64, SE = 18.23, *p* = 0.017), and cognitive flexibility had a significant predictive effect on divergent thinking (*b* = −0.45, SE = 0.01, *p* < 0.001). The indirect effect of cognitive flexibility was 0.19, and its 95% confidence interval was [0.037, 0.394]. The indirect effect accounted for 45.7% of the total effect (*b* = 0.41, SE = 0.17, *p* = 0.018).

## Discussion

There have been mixed results regarding whether bilingualism is associated with creativity. Our results showed that on average, high-proficient Chinese-English bilinguals had higher convergent and divergent thinking than low-proficient bilinguals. More importantly, we found that the association between bilingualism and creativity was mediated by cognitive control. The results supported our hypothesis that cognitive inhibition would mediate the association between bilingualism and convergent thinking, and cognitive flexibility would mediate the association between bilingualism and divergent thinking. This study provides the strong evidence to document the role of cognitive control in explaining why bilingualism has been linked to creativity in many studies.

The present study found that bilingualism was significantly associated with convergent thinking and divergent thinking, suggesting a bilingual advantage on creativity (Sampedro and Peña, 2019; Leikin et al., 2020). The positive relationship between bilingualism and convergent thinking was documented to be relatively stable in previous studies (Hommel et al., 2011). However, several studies which focused on divergent thinking found the controversial relationship between bilingualism and creativity (Hommel et al., 2011; Lange et al., 2020; Booton et al., 2021). The present study adopted a figural creativity task rather than a verbal creativity task, and found a significant positive association with bilingualism. This finding was consistent with those of previous studies (Sampedro and Peña, 2019).

The results showed that bilingualism was significantly, positively associated with cognitive inhibition and cognitive flexibility, consistent with previous findings. Bilinguals often need to bias the competition from different linguistic representations. This requires cognitive inhibition by top–down processes according to individual goals, which is likely to promote cognitive inhibition capacity (Hommel et al., 2011). Meanwhile, having to switch between two language requires cognitive flexibility, and thus bilinguals might develop greater cognitive flexibility capacity than monolinguals (Rosselli et al., 2016).

The present study also found that cognitive inhibition and cognitive flexibility were respective mediators in the associations between bilingualism and the two elements of creativity. With regard to cognitive inhibition as a mediator, we found evidence of both parts of the mediated pathway, namely the link between bilingualism and cognitive inhibition and in turn, the link between cognitive inhibition and convergent thinking. Several scholars have raised doubts about the presence and importance of the first link in the proposed mediated process (Poulisse and Bongaerts, 1994; Colzato et al., 2008). Colzato et al. (2008) compared monolinguals and bilinguals with regard to stop signal performance, inhibition of return, and the attentional blink, and found that bilinguals do not differ from monolinguals in terms of active inhibition but have acquired a better ability to maintain action goals and to use them to bias goal-related information. The argument is that learning multiple languages does not improve inhibition, but leads to stronger and more selective cognitive control (Colzato et al., 2008). Indeed, some studies that have documented natural bilinguals’ significant advantages in inhibitory control have also shown advantages in selective attention (Bialystok and Martin, 2004; Bialystok et al., 2007; Kharkhurin, 2011).

In addition, the results in the present study indicated that cognitive flexibility mediated the association between bilingualism and divergent thinking. These results are consistent with Kharkhurin’s (2017) study, in which bilinguals outperformed their monolingual counterparts on the divergent thinking trait of cognitive flexibility. Second language learners may be immersed in different cultural environments with different concepts and norms, perhaps enriching their conceptual system and associative ability. Indeed, some researchers assert that bilinguals’ enhanced divergent thinking is merely a result of richer experience (Kharkhurin, 2010b). However, associative ability is a necessary prerequisite for divergent thinking, and individuals are more likely to generate divergent thinking when they have many ideas (Nijstad et al., 2010; Chen et al., 2019).

Hommel et al. (2011) showed that on average, high-proficient bilinguals had lower scores on the AUT than low-proficient bilinguals on average. In their study, language proficiency was positively correlated with focused attention and selective attention, skills that are positively related to convergent thinking, instead of divergent thinking. However, Hommel et al. (2011) findings were significant only for fluency part and should not be taken as evidence that bilingualism does not promote divergent thinking. By contrast, most of the earlier studies investigating the relationship between bilingualism and creativity in children have found that high-proficiency bilinguals have a significant advantage in various verbal and non-verbal tests of divergent thinking (Garcia, 1996). In addition, cognitive flexibility has been shown to be positively correlated with divergent thinking (Zabelina and Ganis, 2018; Palmiero et al., 2022). Therefore, we conclude that cognitive flexibility is likely to mediate the relationship between bilingual proficiency and divergent thinking.

Our results suggest that bilingual learning enhances convergent thinking through cognitive inhibition, and enhances divergent thinking through cognitive flexibility. Both possibilities have been proposed (Dijk et al., 2019), but researchers tend to emphasize one aspect of creative thinking (convergent or divergent think) while ignoring the other. The present study systematically examined the relationships among bilingual proficiency, cognitive development, convergent thinking and divergent thinking, suggesting that bilingual learning can improve convergent thinking by promoting cognitive inhibition, and also can improve divergent thinking by promoting cognitive flexibility.

It is worth noting that a large number of studies on the relationship between bilingualism and creativity have been conducted with child samples (Sampedro and Peña, 2019; Booton et al., 2021). The advantage of studying children is that adults’ cognitive functions have already been formed and the standard tests of cognitive function are likely not sensitive enough to detect possible differences (Leikin et al., 2020). The participants in the present study were all university students, and the group differences that were found were likely to be affected by some other factors (e.g., culture and teaching methods). Recent studies found that bilingual college students usually had more accessible knowledge of cultures (Ritter et al., 2012), and generally demonstrated higher levels of open-mindedness and cognitive flexibility (Kubota et al., 2020). Samuel et al. (2018) found that the bilinguals who grown up in an East Asian culture were likely to report significant bilingual advantage, and nine of the ten experiments where the monolinguals spoke a European language (usually English) and the bilinguals spoke either Chinese (including from Hong Kong), Japanese, or Korean. These personality traits have been found to be beneficial to creativity (Chen et al., 2022). Future research is necessary to explore the interplay among culture, education, and bilingualism in relation to creativity.

Our research provides empirical evidence for the relationship and mechanism between bilingual learning and creativity. However, the study has limitations that need to be taken into account. A self-assessment scale and the National English Test for College Students (CET-4 and CET-6 levels) were used to measure bilingualism. It is suggested that future studies can further verify the results of the current research by using actual language proficiency tests. In addition, small sample sizes are likely to reduce the likelihood of finding a genuine effect (Booton et al., 2021), and the cross-sectional design of this study precludes inferences about directionality. Future studies should increase the sample size to obtain more reliable results and conduct longitudinal study to find a causal relationship. Cultural diversity is another important factor that could affect estimations of the link between bilingualism and creativity, and it will be worthwhile to examine whether the results of this study of Chinese-English bilinguals will generalize to other bilingual speakers.

## Data availability statement

The raw data supporting the conclusions of this article will be made available by the authors, without undue reservation.

## Ethics statement

The studies involving human participants were reviewed and approved by Academic Ethics Committee of Guangdong University of Technology. The participants provided their written informed consent to participate in this study. The patients/participants provided their written informed consent to participate in this study.

## Author contributions

TX and JG designed the study. YA and TX collected and analyzed the data. TX, YA, and JG wrote the first draft of the manuscript. All authors contributed to the article and approved the submitted version.

## Funding

This research was funded by grants from the National Social Science Foundation of China (18BYY089).

## Conflict of interest

The authors declare that the research was conducted in the absence of any commercial or financial relationships that could be construed as a potential conflict of interest.

## Publisher’s note

All claims expressed in this article are solely those of the authors and do not necessarily represent those of their affiliated organizations, or those of the publisher, the editors and the reviewers. Any product that may be evaluated in this article, or claim that may be made by its manufacturer, is not guaranteed or endorsed by the publisher.
